# Acute hepatic porphyria masquerading as familial Mediterranean fever: results of a cross-sectional porphobilinogen screening

**DOI:** 10.1186/s13023-026-04308-3

**Published:** 2026-03-19

**Authors:** Gulustan Musayeva, Esra İşat, Mehmet Yıldız, Mehmet Şerif Cansever, Serdal Uğurlu, Dilek Uludağ Alkaya, Sezgin Şahin, Kenan Barut, Ertuğrul Kıykım, Çiğdem Aktuğlu-Zeybek, Özgür Kasapçopur, Tanyel Zubarioglu

**Affiliations:** 1 Department of Pediatrics, İstanbul University-Cerrahpaşa, Cerrahpaşa Medical Faculty, İstanbul, Turkey; 2 Research Laboratory of Metabolism, Cerrahpaşa Medical Faculty, İstanbul-University-Cerrahpaşa, İstanbul, Turkey; 3 Department of Pediatric Rheumatology, Cerrahpaşa Medical Faculty, İstanbul-University-Cerrahpaşa, İstanbul, Turkey; 4 Department of Medical Laboratory Techniques, The Vocational School of Health Services, İstanbul University-Cerrahpaşa, İstanbul, Turkey; 5 Department of Rheumatology, Cerrahpaşa Medical Faculty, İstanbul University-Cerrahpaşa, İstanbul, Turkey; 6 Department of Pediatric Genetics, Cerrahpaşa Medical Faculty, İstanbul-University-Cerrahpaşa, İstanbul, Turkey; 7 Department of Pediatric Nutrition and Metabolism, Cerrahpaşa Medical Faculty, İstanbul University- Cerrahpaşa, İstanbul, Turkey

**Keywords:** Porphyrias, Hepatic, *MEFV*, Familial Mediterranean fever, Porphobilinogen

## Abstract

**Background:**

Acute hepatic porphyria (AHP) is a heme metabolism disorder characterized by acute abdominal pain episodes, with diagnostic delays and misdiagnosis due to heterogeneous presentations and symptom overlap with other diseases. Timely diagnosis is crucial as untreated attacks can lead to life-threatening complications such as hepatocellular carcinoma, chronic kidney disease, neuropathy and chronic pain. AHP often mimics various conditions such as lead poisoning, Guillain-Barré syndrome, vasculitis and surgical abdominal conditions, leading to diagnostic challenges and inappropriate treatment. Familial Mediterranean fever (FMF), another disease with recurrent abdominal pain as its main feature, has a similar symptomatology to AHP. The aim of this study was to investigate the prevalence of AHP in patients with clinically suspected FMF without a confirmatory genotype using cross-sectional urinary porphobilinogen (PBG) screening during abdominal pain episodes.

**Results:**

This cross-sectional study included a total of 104 patients under rheumatology follow-up for suspected FMF and two control groups (genetically confirmed FMF patients and healthy controls). Patients presenting with severe abdominal pain and had a spot urinary PBG/creatinine ratio of ≥ 10 µmol/mmol were diagnosed with AHP together with at least one clinical manifestation defined by the European Porphyria Network. Among 104 suspected-FMF patients, 5 (4.8%) were diagnosed with AHP, with a diagnostic delay of 16.4 ± 10.38 years. AHP patients frequently exhibited neurological (muscle weakness, paresthesia), psychiatric, and gastrointestinal (nausea, vomiting) symptoms. In likelihood ratio analyses, urinary incontinence, hypertension and neuropathic symptoms were strong discriminative indicators of AHP, whereas fever, arthritis, rash and chest pain were more indicative of FMF, aiding differential diagnosis.

**Conclusions:**

Due to diagnostic challenges and overlapping symptoms, AHP should be considered in the differential diagnosis of FMF, particularly in patients with neurological or systemic features. Urinary PBG screening and identifying predictive markers may improve diagnostic accuracy and early management of AHP.

**Supplementary Information:**

The online version contains supplementary material available at 10.1186/s13023-026-04308-3.

## Background

Porphyrias are inherited metabolic disorders caused by defects in heme biosynthesis. They are divided into two subgroups, hepatic and erythropoietic porphyrias. Acute hepatic porphyrias (AHP) are characterized by severe abdominal pain, peripheral neuropathy and autonomic symptoms and include aminolevulinic acid dehydratase (ALAD) deficiency porphyria, acute intermittent porphyria (AIP), hereditary coproporphyria (HCP) and variegate porphyria (VP) [1–4].

The clinical presentation of AHPs is characterized by neurovisceral attacks, with three main components. Severe abdominal pain is the most common symptom and typically the first sign of an attack. Peripheral neuropathy usually develops after the onset of pain, although paresis may occur and progress in advanced cases. Central and autonomic nervous system involvement may cause seizures, psychosis, insomnia and anxiety [3, 5]. Symptoms often develop after puberty and the disease is more common in women. Environmental and metabolic factors, nutrition, and medication, hormone preparations, smoking and fasting can trigger acute attacks [6, 7].

Delayed or missed diagnosis are major challenges in managing AHP mainly due to its heterogeneous clinical presentation and symptom overlap with other diseases [8–10]. Diagnosis is further complicated when neurological symptoms occur without abdominal pain, as many patients experience only a few lifetime attacks [11, 12]. Laboratory challenges also contribute; urinary porphobilinogen (PBG), the key diagnostic test, often yields normal results outside acute attacks and and requires precise timing and careful analytical procedures [3, 13, 14].

Acute hepatic porphyria can mimic lead poisoning, Guillain-Barré syndrome (GBS), vasculitis and polymyositis leading misdiagnosis [8–10]. Familial Mediterranean fever (FMF) is an autosomal recessive autoinflammatory disease characterized by recurrent episodes of fever and serosal inflammation, with abdominal pain as one of the characteristic symptoms. Despite this strong symptomatic overlap, particularly with acute abdominal pain, no studies to date have investigated the prevalence of AHP in FMF patients. To date, only one case has been reported in which a patient previously diagnosed with FMF and treated with colchicine was later diagnosed with AIP [15]. This study was based on the hypothesis that AHP may be underdiagnosed in patients with clinical suspicion of FMF due to overlapping symptoms, especially recurrent abdominal pain. Therefore, we aimed to investigate the prevalence of AHP in this population through cross-sectional quantitative urinary PBG screening during abdominal pain episodes.

## Methods

### Participants

This cross-sectional study was conducted between December 2023 and November 2024.

*The study group* consisted of patients with clinical suspicion of FMF who met the inclusion criteria as follows:

- Patients ≥ 14 years.
- Being under follow-up at a rheumatology center with a clinical suspicion of FMF,
- Presence of severe episodic abdominal pain,
- And having at least one of the following conditions that are not typical for FMF:
  - Lack of a confirmatory *MEFV* genotype,
  - Atypical episode duration for FMF (lasting < 6 h or > 72 h),
  - Pain episodes triggered by specific factors, including hormonal changes (e.g., menstrual cycle, use of oral contraceptives), infections, prolonged fasting, or medication use.

The study also included two control groups:

1. *The diseased control group*, comprising FMF patients with a confirmatory *MEFV* genotype and typical clinical findings of FMF.
2. *The healthy control group*, consisting of age- and sex-matched healthy individuals.

**Fig. 1:**
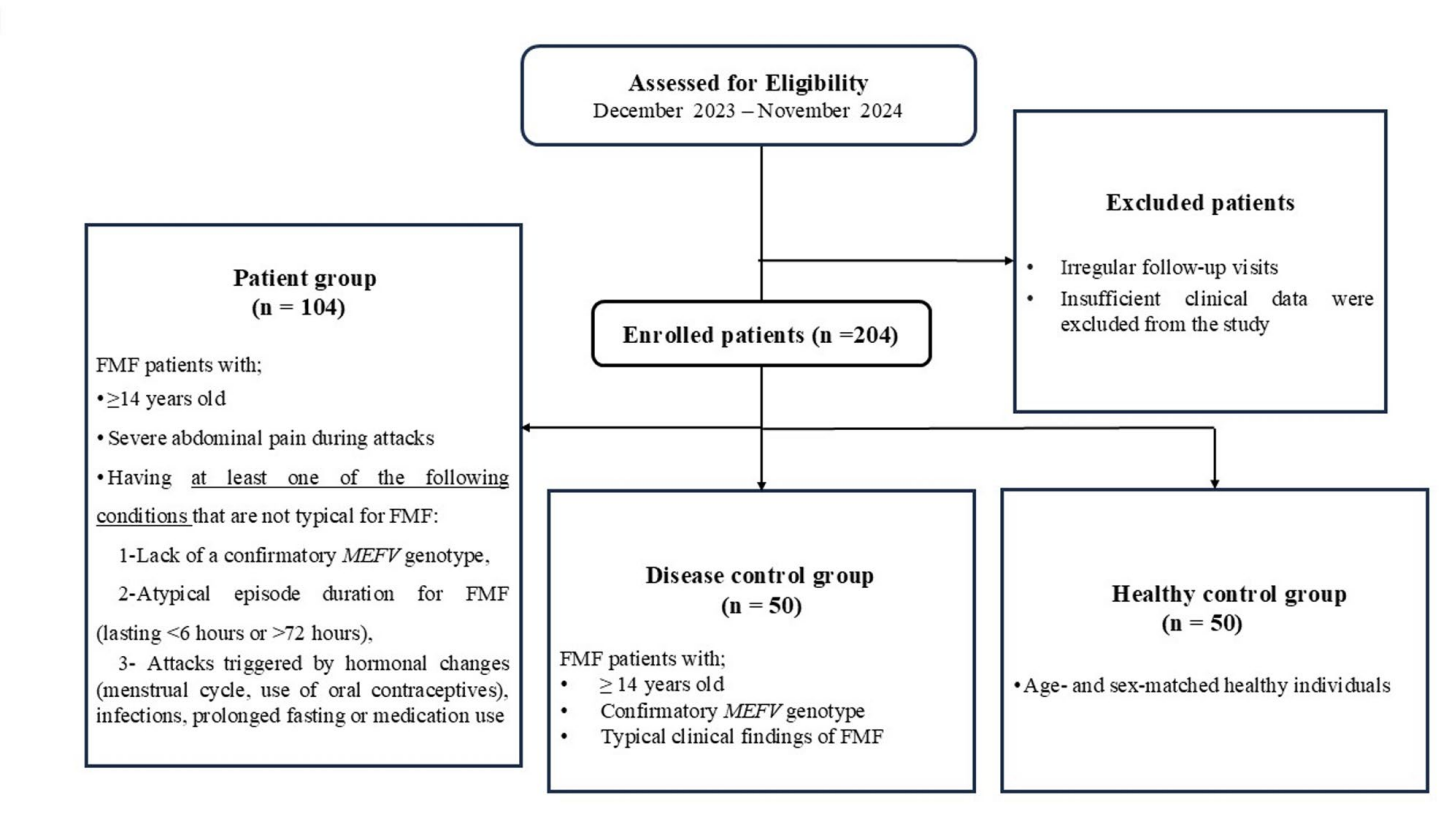
The inclusion and exclusion criteria and the procedure for selecting participants

Patients who did not attend regular follow-up or had insufficient clinical data were excluded. The inclusion and exclusion criteria and procedure for selecting participants are shown in Fig. 1.

Written informed consent was obtained from all participants and the study protocol was approved by the local ethics committee (E-83045809-604.01.01-727609).

### Study design

**Fig. 2:**
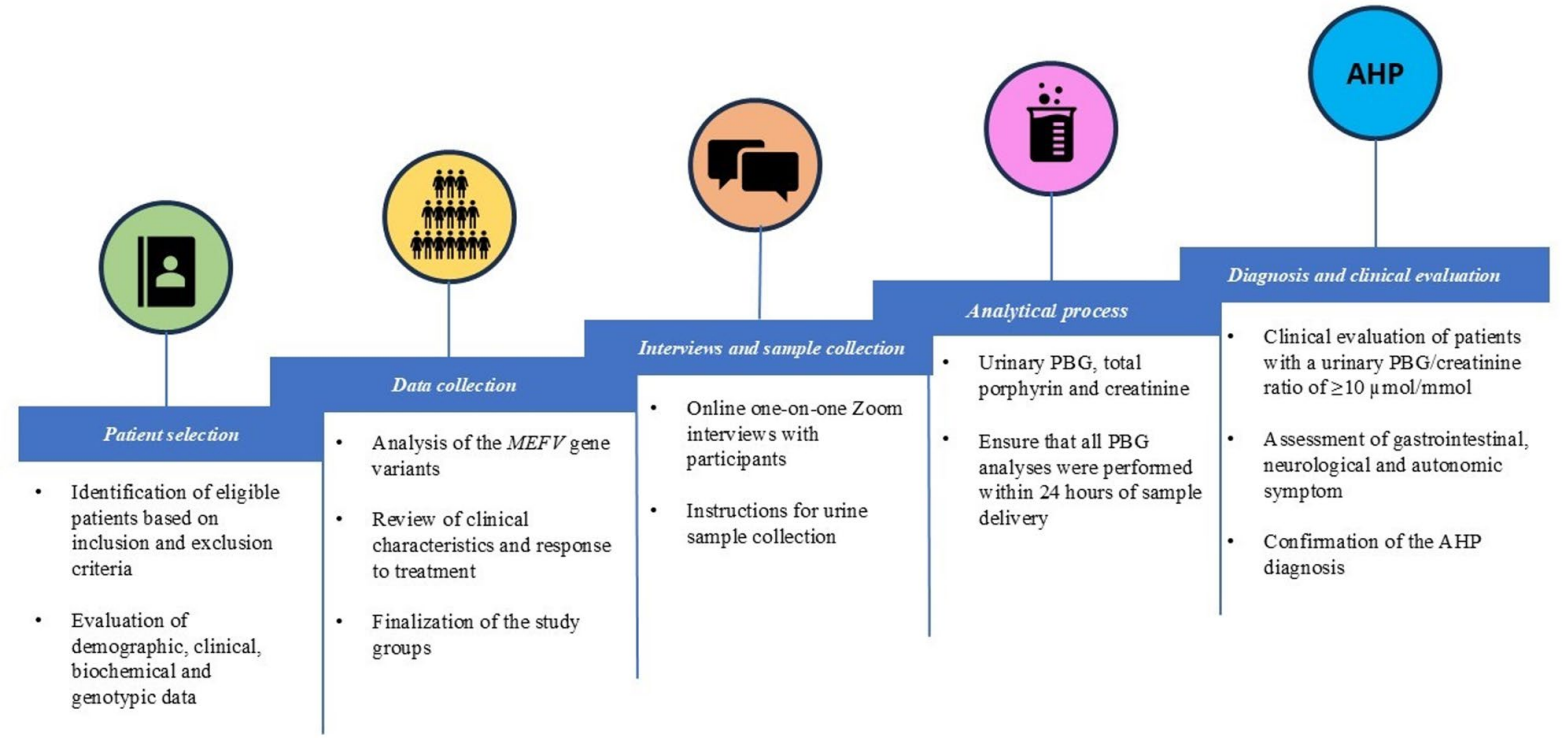
The overall design and schedule of the study

The study was conducted in a sequential approach that began with the identification and grouping of eligible participants based on the inclusion criteria. Subsequently, spot urine samples were collected from participants during abdominal pain episodes, which were properly stored and transported to ensure accuracy and reliability of results. Urine samples were then analyzed for PBG, total porphyrins and creatinine levels, and patients with a urinary PBG/creatinine ratio of ≥ 10 µmol/mmol creatinine underwent a detailed clinical examination to confirm the diagnosis of AHP based on the European Porphyria Network definitions. The overall design and schedule of the study are shown in Fig. 2.

## Sample collection

At the beginning, demographic, clinical, biochemical and genotypic data were evaluated in the rheumatology department. Following data were analyzed: sex, molecular analysis of the *MEFV* gene, age at disease onset and diagnosis, clinical manifestations during attacks, neurological involvement, comorbidities, treatments and treatment responses. Based on these data, patients were assigned to the study or diseased control group.

Online one-to-one interviews were conducted for all participants via Zoom. During these sessions, patients were instructed to take a spot urine sample immediately if they experienced abdominal pain of any kind after being enrolled in the study. They were informed that the urine sample should contain the following;

- Have a volume of at least 10 ml,
- Be protected from light (either stored in opaque containers or wrapped in aluminum foil), and.
- Be stored immediately at + 4 °C.

Patients were also instructed to send the urine sample to the Research Laboratory of the Department of Pediatric Nutrition and Metabolism, within 24 h, adhering to cold chain protocols. Each sample was analyzed for urinary PBG, porphyrins and creatinine. PBG/creatinine and total porphyrins/creatinine ratios were evaluated to standardize the measurements.

### Procedures

The urine samples delivered to the laboratory were immediately divided into two sterile 5 mL containers. All aliquots were wrapped in aluminum foil to protect them from light. The aliquot for PBG analysis was analyzed within 24 h of being received by the laboratory to ensure accuracy and reliability of the results, without being frozen, as PBG levels may decrease with prolonged storage.

The second aliquot, intended for total porphyrin analysis, was immediately frozen at -20 °C and stored until analysis. Prior to porphyrin analysis, the frozen samples were thawed and brought to room temperature.

### Chemical reagents

The chemical reagents used in this study included creatinine, sodium hydroxide (NaOH), picric acid, acetylacetone (AA), ethanol, formaldehyde, perchloric acid (HClO4) and acetic acid (HAc), all of which were purchased from Sigma-Aldrich Química, S.A. (Madrid, Spain). All chemicals were of analytical grade.

### Urine creatinine analysis

Urine creatinine analysis was performed using the Jaffe alkaline picrate method. In this procedure, creatinine in urine reacts with picric acid in an alkaline medium to form the orange-coloured tautomer of creatinine picrate. Absorbance of the reaction product was measured at 540 nm using a Shimadzu UV-1900 UV-Vis spectrophotometer. Results were then calculated accordingly [16–19].

### Urinary porphobilinogen analysis

Urinary PBG analysis was performed using the commercial “ClinEasy^®^ Complete Kit for Porphobilinogen in Urine” (order number 17300) from RECIPE. The method demonstrated a linearity range of 1–5000 µg/L, limit of detection (LOD) of 0.5 µg/L, lower limit of quantitation (LLOQ) of 1 µg/L and a recovery rate of 94–105%. Samples were prepared according to the extraction procedure of the kit, and the absorbance was measured at 553 nm using a Shimadzu UV-1900 UV-Vis spectrophotometer. Results were calculated based on the specifications of the kit.

### Urinary porphyrin analysis

Urinary porphyrin analysis was performed using the “ClinRep^®^ Complete Kit for Porphyrins in Urine”, which includes the analytical method, column and extraction procedure, all supplied by RECIPE. The method demonstrated a linearity range of 0.8–80 mg/L, LOD of 0.3 mg/L, LLOQ of 0.8 mg/L and a recovery rate of 87%. High performance liquid chromatography (HPLC) was performed using the Shimadzu LC-2040 system. Fluorescence detection (FLD) was performed using the system’s RF-20 A fluorescence detector operating at excitation/emission wavelengths of 394/624 nm to ensure optimal sensitivity and accuracy.

### Diagnosis of AHP based on clinical and laboratory findings

Clinical and laboratory characteristics were evaluated based on the definitions of the European Porphyria Network [20]. Patients with a urinary PBG/creatinine ratio of ≥ 10 µmol/mmol creatinine in spot urine samples were interviewed regarding clinical symptoms that occurred during their current or previous abdominal pain episodes. The following symptoms were assessed:

- Gastrointestinal findings: nausea, vomiting and/or constipation.
- Hypertension and/or tachycardia.
- Peripheral neuropathy.
- Urinary dysfunction: retention or incontinence.
- Neuropsychiatric involvement.

Patients presenting with at least one of the above clinical manifestations in addition to a severe abdominal pain attack and a spot urinary PBG/creatinine ratio of ≥ 10 µmol/mmol creatinine were diagnosed with AHP.

### Statistical analysis

Statistical analyses were performed using Statistical Package for Social Sciences version 22.0 (SPSS Inc., Chicago, IL, USA). Descriptive analyses were displayed as mean±standard deviation or median (25th; 75th percentiles) according to distribution. The categorical variables were presented as number (percentages). The normal distribution of data was evaluated with a Kolmogorov–Smirnov test. Categorical variables were analyzed using the chi-square test or Fisher’s exact test, as appropriate based on the expected cell counts. For non-normally distributed data, the Kruskal-Wallis test was used for comparisons involving more than two groups. For normally distributed quantitative variables, comparisons between two groups were performed using the independent samples t-test. A value of *p* < 0.05 was considered statistically significant.

The diagnostic performance of selected clinical features was evaluated by calculating sensitivity, specificity, positive likelihood ratio (LR+) and negative likelihood ratio (LR-), each with the corresponding 95% confidence intervals (CIs). The CIs for the proportions were calculated using the Wilson score method, while the CIs for the likelihood ratios were estimated using the log method. In cases where the contingency tables contained zeros, the Haldane-Anscombe correction was applied by adding 0.5 to each cell to allow for a valid calculation.

## Results

A total of 204 participants were enrolled in the study, including 104 (50.98%) patients who met the inclusion criteria for the study group, 50 (24.51%) FMF patients with confirmatory *MEFV* genotype in the diseased control group and 50 (24.51%) healthy volunteers as the healthy control group.

In the study group, 43 (41.35%) were male and 61 (58.65%) were female. The median age of the patients was 19 (17;21) years. Baseline characteristics of the groups are given in Table 1.

**Table 1:** Comparison of clinical and treatment characteristics between the study group (clinical suspicion of FMF) and diseased control group (FMF patients with confirmatory genotype) enrolled in the study

Age (years)	Study Group (*n* = 104) Patients with clinical suspicion of FMF	Diseased Control Group (*n* = 50) FMF patients with confirmatory genotype	*p*
	19.8 ± 8.26	26.96 ± 12.82	0.001^*^
**Sex (n)**	Female,61 Male,43	Female,27 Male,23	0.585^**^
**Clinical characteristics (%)**			
*Abdominal pain*	75.9	88	0.081^**^
*Muscle weakness*	32.6	14	**0.014** ^**^
*Myalgia*	100	56	**0.000** ^**^
*Arthralgia*	65.3	68	0.748^**^
*Arthritis*	0	60	**0.000** ^**^
*Headache*	36.5	24	0.120^**^
*Vomiting*	10.5	8	0.614^**^
*Nausea*	29.8	20	0.197^**^
*Diarrhea*	20.1	24	0.590^**^
*Constipation*	15.3	22	0.312^**^
*Urinary incontinence*	2.8	4	0.660^***^
*Urinary retention*	3.8	0	0.305^***^
*Seizure*	1.9	2	1.00^***^
*Neuropathic symptoms*	3.8	0	0.551^***^
*Psychiatric changes*	25.9	6	**0.003** ^**^
*Hypertension*	3.8	0	0.305^***^
*Fever*	33.7	40	0.442^**^
*Rash*	0	16	**0.000** ^**^
*Chest pain*	5.8	44	**0.000** ^**^
*Family history of FMF*	48.1	66	**0.040** ^**^
**Comorbidities**	Allergic rhinitis Allergic asthma Acute rheumatic fever Splenomegaly Migraine Epilepsy Proteinuria Henoch-Schönlein purpura Hypothyroidism PFAPA syndrome	Allergic asthma Hypophysis adenoma Migraine Aortic stenosis	
**Treatments**			
Use of Colchicine (n)	83	50	
Use of Biological Agents (n)	0	0	

* Independent samples t-test **Chi-square test *** Fisher’s exact test

*FMF*,* familial Mediterranean fever; PFAPA Syndrome*,* Periodic Fever*,* Aphthous Stomatitis*,* Pharyngitis*,* and Adenitis*

### Comparison of clinical and treatment characteristics between the study group (clinical suspicion of FMF) and diseased control group (FMF patients with confirmatory genotype)

Comparison of clinical characteristics between study group and diseased control group revealed significant differences in certain symptoms. Muscle weakness (*p* = 0.014), myalgia (*p* < 0.001) and psychiatric changes (*p* = 0.003) were significantly more frequent in the study group. In contrast, arthritis (*p* < 0.001), rash (*p* < 0.001) and chest pain (*p* = 0.000) were significantly more frequent in the diseased control group, whereas they were only rarely observed in the study group. A family history of FMF (*p* = 0.040) was also more common in genetically confirmed FMF patients. There were no statistically significant differences in common FMF-related symptoms such as abdominal pain, arthralgia, headache and gastrointestinal disturbances (nausea, vomiting, diarrhea, constipation). Rare symptoms including urinary dysfunction, neuropathic symptoms and seizures were also comparable between the groups.

Regarding treatment, colchicine was frequently used in both groups (83 patients in the patient group and 50 in the control group), while none of the participants required biologic agents.

**Fig. 3:**
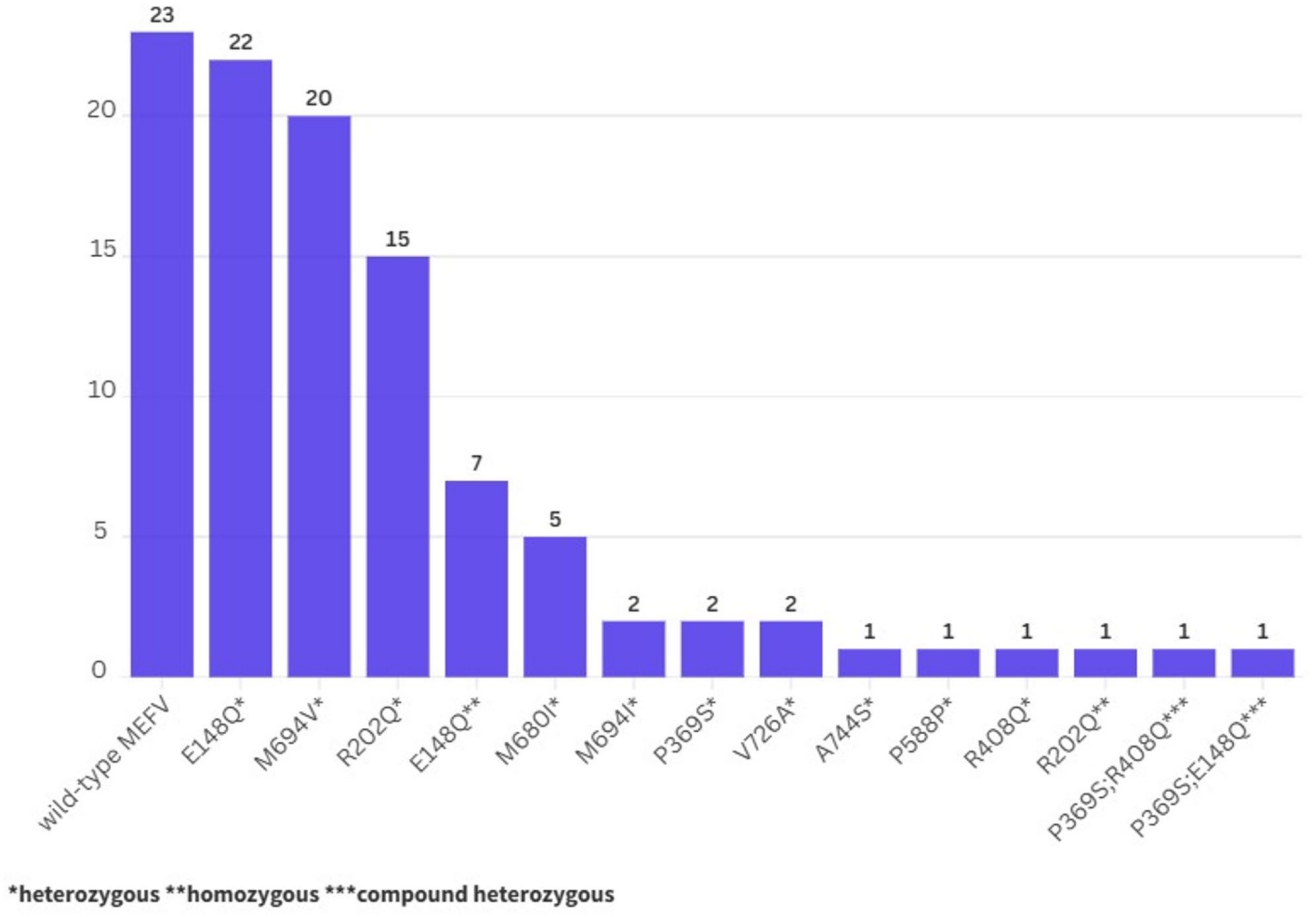
The molecular analysis of the distribution of the *MEFV* gene for the study group

Detailed comparison of demographic and clinical characteristics between two groups is shown in Table 1. Molecular analysis of the distribution of the *MEFV* gene for the study group is also shown in Fig. 3.

### Evaluation of the urinary PBG/creatinine analysis

All participants in both control groups had a urinary PBG/creatinine ratio below 10 µmol/mmol creatinine. The median urinary PBG/creatinine levels were 0.59 (0.27; 0.93) µmol/mmol in diseased control group and 0.47 (0.28; 0.81) µmol/mmol in healthy control group, with no significant difference between groups (*p* > 0.05).

In the study group, five (4.8%) had urinary PBG/creatinine ratio above 10 µmol/mmol, while the remaining 99 patients had values below this threshold, with a median ratio of 0.63 (0.37; 1.06) µmol/mmol creatinine. After excluding five AHP-diagnosed patients, no statistically significant difference in urinary PBG/creatinine levels was observed among the three groups (*p* > 0.05). Urinary PBG/creatinine data of the participants, excluding patients diagnosed with AHP, are shown in Supplementary file 1.

### Demographic, clinical and biochemical characteristics of the AHP patients

Five patients with a urinary PBG/creatinine ratio of ≥ 10 µmol/mmol were examined for clinical signs of an acute porphyria attack. As all had at least one clinical feature defined by the European Porphyria Network, in addition to severe abdominal pain, they were diagnosed with AHP.

Among these five AHP patients, females predominated (4:1). Molecular analysis of the *MEFV* gene revealed the R202Q variant (heterozygous) in three patients (60%) and the M694I variant (heterozygous) in one patient, while one patient had a wild-type *MEFV* analysis. The mean age was 22.82 ± 9.59 years, with initial symptoms appearing at the age of 8.8 ± 2.94 years, resulting in a diagnostic delay of 16.4 ± 10.38 years. Three AHP patients (60%) reported partial symptom relief with colchicine.

Symptoms of the acute attack varied, although myalgia and muscle weakness were consistently present. Gastrointestinal symptoms were observed in four patients, while paresthesia (*n* = 4) was another common finding. Urinary incontinence (*n* = 2), headache (*n* = 2), psychiatric changes (*n* = 4) and hypertension (*n* = 2) were less frequent.

Demographic, clinical and biochemical characteristics of patients diagnosed with AHP are summarized in Table 2.

**Table 2:** Demographic, clinical and biochemical characteristics of patients diagnosed with acute hepatic porphyria (*n* = 5)

	Case 1	Case 2	Case 3	Case 4	Case 5
***Demographic characteristics***					
Sex	F	F	M	F	F
*MEFV* gene analysis	M694I het	R202Q het	R202Q het	R202Q het	Normal
Age of onset (years)	12	12	7	7	6
Age of AHP diagnosis	46	21	20	16	23
Diagnostic delay (years)	34	9	13	9	17
Patient-reported symptom relief to colchicine treatment	No	Yes	Yes	Yes	No
***Clinical findings during attacks***					
Abdominal pain Myalgia	+ +	+ +	+ +	+ +	+ +
Headache	+	+			
Paresthesia		+	+	+	
Muscle weakness	+	+	+	+	+
Urinary incontinence			+		+
Nausea	+	+	+		+
Vomiting	+		+		+
Diarrhea			+		+
Constipation			+		+
Psychiatric changes Hypertension	+ +	+ +	+	+	
***Acute inflammatory markers*** ^*^					
WBC (10^3^/µL, N:4–10 10^3^/µL)	8.80	10.28	8.60	9.30	8.10
CRP (mg/L, N:5 mg/L)	22.09	1.54	8.60	9.57	0.58
***Porphyria biomarkers***					
PBG/creatinine (µmol/mmol creatinine)	10.37	33.51	14.12	10.77	19.77
UROI/creatinine (nmol/mmol creatinine)	1.97	0.66	1.00	0.07	1.19
HEPTA I/creatinine (nmol/mmol creatinine)	0.80	0.23	0.36	0.03	0.34
HEXA/creatinine (nmol/mmol creatinine)	0.19	2.25	0.19	0.19	0.57
PENTAI/creatinine (nmol/mmol creatinine)	0.15	3.75	0.21	0.27	0.69
COPROI/creatinine (nmol/mmol creatinine)	7.85	4.74	2.69	1.38	3.19
COPROIII/creatinine (nmol/mmol creatinine)	18.09	7.19	9.88	1.96	7.36
Total Porphyrin (nmol/mmol creatinine)	29.07	18.84	14.35	3.93	13.35

^*^ Acute inflammatory markers were retrospectively obtained from the medical records of patients during a symptomatic period before treatment initiation

*M*,* male; F*,* female; AHP*,* acute hepatic porphyria; FMF. familial Mediterranean fever; PBG*,* porphobilinogen; UROI*,* uroporphyrin I; UROIII*,* uroporphyrin III; HEPTAI*,* heptacarboxylic porphyrin; HEXA*,* hexacarboxylic porphyrin; PENTAI*,* pentacarboxylic porphyrin; COPROI*,* coproporphyrin I; COPROIII*,* coproporphyrin III; WBC*,* white blood cells; CRP*,* C-reactive protein*

### Detailed medical history of AHP patients

The clinical and diagnostic features of the five patients diagnosed with AHP are presented below.

#### Case 1

A 46-year-old female patient presented with severe abdominal pain episodes that first occurred at the age of 12 during her menstruation. Neurologic and psychiatric symptoms occurred during the episodes, including headache, leg pain, muscle weakness, nausea/vomiting, and anxiety. Genetic analysis revealed the M694I heterozygous variant in the *MEFV* gene, which led to the suspicion of FMF, and colchicine therapy was initiated; however, symptoms persisted despite treatment.

The patient had several hospitalizations for chronic health problems. She was diagnosed with migraine due to chronic headaches that lasted 24 h and limited physical activity, but did not respond to amitriptyline or flunarizine therapy. Intermittent hypotension and hypertension were diagnosed on headache examination and antihypertensive treatment was initiated. Two years prior to the study, the patient was diagnosed with Helicobacter pylori infection after complaining of nausea, vomiting and a 10 kg weight loss; treatment was ineffective. Neuropathic pain in both lower extremities was investigated, with normal EMG findings and no benefit from symptomatic treatments. At the time of enrollment, the patient was receiving psychiatric treatment for symptoms such as suicidal ideation, panic attacks, anxiety, and crying spells, but treatment outcomes remained limited. Following the diagnosis of AHP, molecular analysis was recommended but could not be performed due to the lack of patient consent.

#### Case 2

A 21-year-old female presented with severe abdominal pain lasting up to three days, accompanied by myalgia, paresthesia in the lower extremities, headache, muscle weakness, nausea and psychiatric symptoms such as anxiety and panic attacks. These episodes, which significantly limited her physical activity and required emergency visits, began at the age of 12. After *MEFV* molecular analysis revealed the heterozygous R202Q variant, she was started colchicine therapy, which resulted in a partial clinical response.

The patient was treated in various departments for chronic complaints. After bilateral temporal headaches that persisted for about a day, she was diagnosed with tension-type headache and was observed without treatment. During these episodes, intermittent hypertension was detected. For the past two years, she suffered from paresthesia and episodic muscle weakness, for which she was referred to physiotherapy and received exercise recommendations, which did not result in significant improvement. Following the diagnosis of AHP, a multigene panel analysis targeting AHP subtypes was performed and the identified variant was subsequently confirmed by Sanger sequencing. The analysis revealed a heterozygous HMBS (NM_000190.4): c.33 + 132 − 33 + 135del variant, resulting in a diagnosis of AIP.

#### Case 3

A 20-year-old male first experienced episodes of abdominal pain, myalgia in the legs and difficulty walking at the age of 7. These attacks, which lasted an average of three days, were accompanied by intermittent constipation, diarrhea, nausea and vomiting and significantly limited his physical activity. During attacks, additional symptoms such as urinary incontinence, muscle weakness, paresthesia and mood alterations such as aggression occurred. Analysis of the *MEFV* gene revealed the R202Q heterozygous variant, and colchicine therapy was initiated. The patient responded well to colchicine treatment. Following the diagnosis of AHP, a multigene panel analysis targeting AHP subtypes was performed and the identified variant was subsequently confirmed by Sanger sequencing. The analysis revealed a heterozygous CPOX (NM_000097.7): c.1278-94del variant, resulting in a diagnosis of HCP.

#### Case 4

A 16-year-old female first presented at the age of 7 years with episodic abdominal pain, pain and paresthesias in the legs. Suspecting FMF, molecular analysis of the *MEFV* gene revealed the R202Q heterozygous variant and colchicine therapy was initiated, which showed a symptom relief. At the time of enrollment in the study, she was undergoing follow-up care for social withdrawal and anxiety that had developed over the previous year. Following the diagnosis of AHP, molecular analysis was recommended but could not be performed due to the lack of patient consent.

#### Case 5

A 23-year-old female, first experienced episodic abdominal and leg pain first occurred at the age of 6 years, lasting an average of three days and limiting physical activity. Symptoms during the attacks included muscle weakness, nausea, vomiting, diarrhea and urinary incontinence. FMF was initially suspected, but the genetic analysis revealed a wild-type *MEFV*. Colchicine therapy was initiated to assess clinical response, but was discontinued due to lack of efficacy. Over the course of the past year, the patient visited the clinic several times due to various complaints. She reported pain in her lower extremities that resulted in limited movement, but diagnostic tests were normal and exercise therapy was of no benefit. She also had constipation, which improved with treatment, although intermittent diarrhea persisted. In addition, she presented with hand tremor, but neurological examinations were unremarkable. Following the diagnosis of AHP, a multigene panel analysis targeting AHP subtypes was performed and the identified variant was subsequently confirmed by Sanger sequencing. The analysis revealed a heterozygous CPOX (NM_000097.7): c.1278-94del variant, resulting in a diagnosis of HCP.

### Possible predictors of AHP in patients with clinical suspicion of FMF

Within the cohort of 104 individuals in the study group, the diagnostic utility of individual clinical features was further evaluated using sensitivity, specificity and LR for predicting the diagnosis of AHP in this group. The data are presented in Table 3.

**Table 3:** Diagnostic performance of clinical features to differentiate AHP from FMF: sensitivity, specificity and likelihood ratios with 95% confidence intervals

Symptom	Sensitivity % (95% CI)	Specificity % (95% CI)	LR+ (95% CI)	LR- (95% CI)
Muscle weakness	100 (48–100)	70.7 (61.7–79.7)	3.41 (2.52–4.64)	0
Myalgia	100 (48–100)	0 (0-3.7)	1 (NA)	∞
Arthralgia	80 (28–99)	35.3 (26-44.8)	1.23 (0.83–1.86)	0.56 (0.09–3.42)
Headache	40 (5–85)	63.6 (54.2–73)	1.1 (0.52–2.34)	0.94 (0.49–1.82)
Vomiting	60 (15–95)	91.9 (87–97)	7.42 (2.8–19.5)	0.43 (0.15–1.27)
Nausea	80 (28–99)	72.7 (63.9–81.5)	2.93 (1.7–5.04)	0.27 (0.05–1.59)
Fever	20 (1–62)	65.6 (56.4–75)	0.58 (0.1–3.44)	1.21 (0.77–1.93)
Diarrhea	40 (5–85)	80.8 (73-88.6)	2.08 (0.92–4.7)	0.74 (0.38–1.43)
Constipation	40 (5–85)	85.8 (78.9–92.7)	2.82 (1.23–6.45)	0.69 (0.35–1.38)
Urinary incontinence	40 (5–85)	98.9 (97–100)	39.6 (4.28–368)	0.60 (0.30–1.24)
Urinary retention	20 (1–62)	96.9 (93.5–100)	6.6 (0.81–51.6)	0.82 (0.53–1.28)
Seizure	0 (0–52)	97.9 (95.2–100)	0	1.02 (0.79–1.31)
Neuropathic symptoms	60 (15–95)	100 (96.4–100)	∞	0.4 (0.14–1.17)
Psychiatric changes	80 (28–99)	76.7 (68.5–85.1)	3.44 (1.96–6.05)	0.26 (0.05–1.51)
Hypertension	40 (5–85)	97.9 (95.2–100)	19.8 (3.46–113)	0.61 (0.30–1.25)
Arthritis	0 (0–52)	100 (96.4–100)	NA	1 (0.82–1.21)
Rash	0 (0–52)	100 (96.4–100)	NA	1 (0.82–1.21)
Chest pain	0 (0–52)	93.9 (88.7–97.1)	0	1.06 (0.96–1.17)
Family history of FMF	40 (5–85)	51.5 (41.7–61.3)	0.82 (0.49–1.37)	1.16 (0.66–2.02)

∞ (infinite) indicates no false positives, LR+ approaches infinity

0 (zero)indicates no false negatives, LR- is zero

NA: Not applicable; calculation not possible due to zero counts in relevant cells

*AHP*,* acute hepatic porphyria; FMF*,* familial Mediterranean fever; CI*,* confidence interval; LR+*,* positive likelihood ratio; LR-*,* negative likelihood ratio*

Symptoms such as vomiting had high specificity and increased LR+, indicating strong discriminative value for identifying AHP in patients with suspected FMF. Notably, neuropathic symptoms were exclusively observed in AHP cases yielding an LR + of ∞, as there were no false positives. Urinary incontinence and hypertension were less common but also had high LR+ values, indicating strong diagnostic relevance.

Conversely, FMF-specific features such as fever, arthritis, rash and chest pain showed high specificity for FMF and were not present in AHP patients. This pattern suggests that their presence may contribute to exclude AHP in the differential diagnosis.

Although certain features had very low LR− values, suggesting that their absence is a potential benefit for ruling out AHP, these results should be interpreted with caution given the very small number of AHP cases and the resulting wide confidence intervals.

## Discussion

In this cross-sectional study, quantitative urinary PBG measurements were performed in 104 patients with clinical suspicion of FMF during an episode of abdominal pain. Based on the urinary PBG/creatinine ratio and clinical findings as defined by the European Porphyria Network, five patients (4.8%) were diagnosed with AHP. Diagnostic delay in these patients was 16.4 ± 10.38 years. Neurological (muscle weakness, myalgia, paresthesia), psychiatric and gastrointestinal (nausea, vomiting, diarrhea, constipation) symptoms were particularly common in AHP patients. In likelihood ratio analyzes, urinary incontinence, hypertension and especially neuropathic symptoms proved to be strong discriminative findings in favor of AHP, while classic FMF-associated features such as fever, arthritis, rash and chest pain were more suggestive of FMF and thus contributed to the differential diagnosis. In addition, patient-reported symptom relief to colchicine was observed in three (60%) of the AHP patients, suggesting a potential role for anti-inflammatory treatments in the management of AHP.

Timely diagnosis of AHP is crucial, as untreated acute and recurrent attacks can lead to hepatocellular carcinoma, chronic kidney disease, peripheral neuropathy, chronic pain and hypertension [3, 21, 22]. Neuropsychiatric symptoms, pancreatitis and cardiac arrhythmias may also develop in patients with recurrent attacks [23]. Delayed diagnosis not only increases complications but also risk of iatrogenic damage, as certain medications prescribed to relieve symptoms can trigger porphyria attacks and exacerbate disease progression. Despite the need for early detection, many patients experience significant diagnostic delays, with studies reporting an average delay of approximately 15 years in the United States and Europe [24]. Another major challenge is misdiagnosis due to symptom overlap. Cases of lead poisoning, appendicitis, and partial bowel obstruction initially mistaken for AHP have been reported [8, 9]. Additionally, motor and axonal neuropathies in AHP can mimic GBS, heavy metal poisoning, paraneoplastic syndromes, severe vitamin deficiencies, diabetes mellitus and drug-induced neuropathies. Previous studies have described cases initially misdiagnosed as GBS, irritable bowel syndrome or NMDA receptor-related autoimmune encephalitis, later correctly diagnosed as AIP or VP [10, 11].

In our study, the diagnostic delay was 16.4 ± 10.38 years. Most AHP patients had a history of multiple hospitalizations for abdominal pain, neuropathic symptoms, headache, and mood disturbances, which often led to misdiagnosis and treatment in different outpatient clinics. Gastrointestinal symptoms occurred in 80% of cases, while myalgias and muscle weakness were observed in all cases. In addition, paresthesia (80%) and psychiatric changes (80%) were common, further complicating the diagnosis. These results highlight the complexity of AHP diagnosis and the need for a comprehensive evaluation, especially in patients with recurrent abdominal pain, neurological symptoms and unexplained systemic findings.

The interaction between AHP and inflammatory diseases remains an important area of research. A key question is whether AHP is masked by underlying inflammation or occurs as a consequence of chronic inflammatory processes. Research suggests a possible pathophysiological link, as symptomatic AIP patients have elevated levels of proinflammatory cytokines, complement, immunoglobulins and vascular endothelial growth factors compared to healthy controls [25].

Case reports indicate an association between AHP and systemic inflammatory diseases, suggesting common pathologic mechanisms. Systemic lupus erythematosus has been associated with AIP since 1952, but only 15 coexisting cases have been reported [26–28]. Similarly, two patients diagnosed with AIP were reported who later developed primary Sjögren’s syndrome [29] and rheumatoid arthritis [30]. These findings highlight the need for further research into the common immunological pathways between AHP and autoimmune diseases and their impact on diagnosis and treatment.

Familial Mediterranean fever is an autosomal recessive autoinflammatory disorder characterized by recurrent fever episodes and serosal inflammation with elevated inflammatory markers [31]. It results from *MEFV* gene variants, which encodes pyrin, an important inflammasomal protein. Mutations in pyrin cause dysregulated inflammasome activity leading to excessive interleukin-1 production, a major proinflammatory cytokine [32]. Diagnosing FMF is challenging due to non-specific symptoms and overlap with other conditions, often causing delayed or incorrect diagnoses, especially in the absence of a definitive diagnostic test. Although *MEFV* gene identification initially raised hopes for an accurate diagnosis, molecular testing alone is insufficient as many clinically diagnosed FMF patients lack biallelic pathogenic variants, while some carriers remain asymptomatic, indicating variable penetrance and additional genetic or environmental influences [33]. Historically, FMF diagnosis was based solely on clinical findings, even without genetic confirmation. However, recent evidence emphasizes the need for caution in diagnosing FMF without confirmatory genotype necessitating thorough differential diagnosis to exclude other autoinflammatory diseases [34]. The current approach emphasizes a combined evaluation of clinical features and *MEFV* variants rather than relying solely on genetic or clinical findings.

Only one case report described coexistence of FMF and AHP. A pregnant woman with fever, weakness, chest and abdominal pain with a homozygous *MEFV* gene variant was treated with colchicine for FMF, but later developed neuropsychiatric symptoms and dark urine, leading to a diagnosis of AIP [15]. In our study, five (4.8%) of 104 FMF patients were diagnosed with AHP. Urinary incontinence and hypertension were significant predictors. These findings suggest that although AHP is considered rare, it may be underdiagnosed.

Colchicine is the first-line treatment for FMF, binding to β-tubulin and causing microtubule depolymerisation, which inhibits cell division and acts as an anti-inflammatory agent [35]. A previous study reported that in four AHP patients receiving colchicine for abdominal pain, administration during prodromal symptoms prevented attacks, while post-onset usage shortened attack duration and reduced pain severity [36]. This symptomatic improvement in AHP patients receiving colchicine may reflect an underlying link between inflammation and the pathophysiology of AHP. Beyond their metabolic basis, AHP - particularly AIP - appear to have systemic inflammatory features that may overlap with FMF. In a case-control study of AIP (*n* = 50), plasma concentrations of IL-1β, TNF-α, IL-6, IL-17 and IFN-γ were significantly higher than in matched control subjects, along with increased complement activation (C3bc and terminal complement complex); within this cohort, IL-17 showed the largest relative fold-increase of the cytokines studied [25]. In parallel, FMF- classically an IL-1β/pyrin inflammasome disorder - was also associated with Th17 release: serum IL-17 is elevated compared to healthy controls both during attacks and in attack-free periods, and peripheral blood mononuclear cells from FMF patients secrete more IL-17, especially in those with frequent attacks and with *MEFV* p.M694V homozygosity [37].In our study, three AHP patients showed a partial symptom relief with colchicine, suggesting a potential role for colchicine and other anti-inflammatory agents in AHP treatment. Within this background, our observation of subjective clinical colchicine responsiveness in some of our AHP cases is mechanistically plausible: colchicine disrupts microtubule dynamics and thereby impedes inflammasome assembly (and downstream IL-1β/IL-18 release) via microtubule centrosome pathways that regulate pyrin; however, the clinical evidence in AHP remains limited [38, 39]. These results highlight the need for further research into its therapeutic implications.

Our study had several limitations. First, molecular analysis could not be performed in two patients due to their refusal, preventing the identification of specific genetic variants. Since the stool tests and plasma fluorescence scans were not performed, AHP subtypes could not be determined in these patients. Second, some important clinical data - such as inflammatory biomarkers during symptomatic and asymptomatic phases - were not consistently available due to the retrospective nature of symptom documentation and variability in clinical records. Another limitation is the lack of supportive objective examinations such as electromyography and urodynamic studies. Finally, the cross-sectional design and small sample size, especially the small number of AHP cases, limited the statistical power and interpretation of likelihood ratios due to wide confidence intervals. However, as the first study to systematically investigate AHP prevalence in patients with suspected FMF, our results represent a preliminary contribution to this field and may serve as a guide for future, more extensive investigations.

## Conclusions

This study emphasizes the importance of considering AHP in the differential diagnosis of patients with suspected FMF, especially in patients with neurological symptoms such as paresthesia, psychiatric changes, muscle weakness and systemic findings. Given the diagnostic overlap and potential for misdiagnosis, clinicians should be alert to warning signs such as urinary incontinence and hypertension, which were identified as significant predictors in our cohort. When AHP is suspected, timely and properly collected urinary PBG testing is critical for an accurate diagnosis. Awareness of these warning symptoms and early multidisciplinary collaboration may prevent years of diagnostic delay and allow for more effective management of AHP, which could reduce long-term complications.

## Supplementary Information

Below is the link to the electronic supplementary material.

Supplementary Material 1

## Data Availability

The data that support the findings of this report are available from the corresponding author upon reasonable request.

## Abbreviations

AHP: Acute hepatic porphyria
ALAD: Aminolevulinic acid dehydratase
AIP: Acute intermittent porphyria
HCP: Hereditary coproporphyria
VP: Variegate porphyria
SIADH: Syndrome of inappropriate antidiuretic hormone secretion
PBG: Porphobilinogen
GBS: Guillain-Barré syndrome
FMF: Familial Mediterranean fever
SPSS: Statistical Package for Social Sciences
LR+: Positive likelihood ratio
LR-: Negative likelihood ratio
CI: Confidence interval
CRP: C-reactive protein
WBC: White blood cell
