# Supplementary material for: Acute hepatic porphyria masquerading as familial Mediterranean fever: results of a cross-sectional porphobilinogen screening
Source: Orphanet J Rare Dis. 2026 Mar 19;21:168. doi: 10.1186/s13023-026-04308-3 (PMC13123016; doi:10.1186/s13023-026-04308-3)
Supplement: Supplementary file 1 — Supplementary Material 1 [file 13023_2026_4308_MOESM1_ESM.docx]

**Supplementary Table S1: Comparison of urinary porphobilinogen/creatinine levels between three groups: the study group without AHP diagnosis and the two control groups**

|  | **Study group without AHP diagnosis**  **(n=99)** | **Diseased control group**  **(n=50)** | **Healthy control group**  **(n=50)** | **P** |
| --- | --- | --- | --- | --- |
| urinary PBG/creatinine (µmol/mmol creatinine) | 0.63 (0.37;1.06) | 0.59 (0.27;0.93) | 0.47 (0.28;0.81) | 0,146 |

Data were presented as median (25^th^; 75^th^ percentile). Quantitative data were analyzed by the Kruskall Wallis test.

*AHP, Acute hepatic porphyrias; PBG, porphobilinogen*
